# Messenger RNA delivery of a cartilage-anabolic transcription factor as a disease-modifying strategy for osteoarthritis treatment

**DOI:** 10.1038/srep18743

**Published:** 2016-01-05

**Authors:** Hailati Aini, Keiji Itaka, Ayano Fujisawa, Hirokuni Uchida, Satoshi Uchida, Shigeto Fukushima, Kazunori Kataoka, Taku Saito, Ung-il Chung, Shinsuke Ohba

**Affiliations:** 1 Department of Bioengineering, The University of Tokyo Graduate School of Engineering, 7-3-1 Hongo, Bunkyo-ku, Tokyo 113-8656, Japan; 2 Laboratory of Clinical Biotechnology, Center for Disease Biology and Integrative Medicine, The University of Tokyo Graduate School of Medicine, 7-3-1 Hongo, Bunkyo-ku, Tokyo 113-0033, Japan; 3 Department of Bone and Cartilage Regenerative Medicine and Department of Sensory and Motor System Medicine, The University of Tokyo Graduate School of Medicine, 7-3-1 Hongo, Bunkyo-ku, Tokyo 113-0033, Japan; 4 Department of Materials Engineering, The University of Tokyo Graduate School of Engineering, 7-3-1 Hongo, Bunkyo-ku, Tokyo 113-8656, Japan

## Abstract

Osteoarthritis (OA) is a chronic degenerative joint disease and a major health problem in the elderly population. No disease-modifying osteoarthritis drug (DMOAD) has been made available for clinical use. Here we present a disease-modifying strategy for OA, focusing on messenger RNA (mRNA) delivery of a therapeutic transcription factor using polyethylene glycol (PEG)-polyamino acid block copolymer-based polyplex nanomicelles. When polyplex nanomicelles carrying the cartilage-anabolic, runt-related transcription factor (RUNX) 1 mRNA were injected into mouse OA knee joints, OA progression was significantly suppressed compared with the non-treatment control. Expressions of cartilage-anabolic markers and proliferation were augmented in articular chondrocytes of the *RUNX1*-injected knees. Thus, this study provides a proof of concept of the treatment of degenerative diseases such as OA by the *in situ* mRNA delivery of therapeutic transcription factors; the presented approach will directly connect basic findings on disease-protective or tissue-regenerating factors to disease treatment.

Osteoarthritis (OA) is a chronic degenerative joint disease caused by an imbalance between synthesis and degradation of cartilage tissues^1^. It is the third most common health problem in the elderly population^2^, and has been ranked as the 11th highest contributor to physical disability globally^3^. Above 80% of people over age 75 in Western countries and above 70% of Americans between the ages of 55 and 70 are affected by OA^4^,^5^. Although there were no discernible changes in the prevalence of OA between 1990 and 2010^3^, the Centers for Disease Control and Prevention (CDC) estimated that the number of OA patients in the US would have doubled by 2020, largely because of the rapidly increasing prevalence of obesity and the elderly status of the “baby boomer ”generation^6^; numerous countries have trends of the increased burden of OA^5^.

OA results in pain and mobility limitations. Current clinical therapeutic strategies are largely palliative, mainly relying on pain killers or anti-inflammatory drugs^7^. Thus, modifications to the structural changes caused by OA have drawn the most attention. However, the development of disease-modifying osteoarthritis drugs (DMOADs) remains a challenge, and although there are several candidate drugs, no DMOAD has yet been made available for clinical use^7^,^8^. Most of the DMOAD candidates are ligands acting on target cells through receptors or inhibitors for cartilage catabolic factors, which act outside of target cells. Given that any biological actions are based on gene transcription, the most direct, effective disease-modifying strategy would be targeting transcription factors that initiate a transcriptional cascade for therapeutic effects within target cells. Accumulating knowledge of cartilage development and the pathophysiology of OA at the cellular or molecular level^9^ supports the intracellular manipulation of gene transcription for OA treatment. Thus, a code that induces the expression of such “therapeutic” transcription factors would be a DMOAD, if it could be delivered to target cells in a clinically relevant manner.

Viral and non-viral vectors have been widely used to express transcription factors *in vivo*. However, the former still have safety concerns, including issues related to insertion into the host genome, while the transfection efficiency of the latter remains quite low, especially in post-mitotic cells^10^. Direct delivery of mRNA into cells has been highlighted as an emerging technology in this context^11^,^12^, since it would directly achieve the expression of proteins of interest without the above concerns associated with viral and non-viral vectors; reports of the reprogramming of fibroblasts via mRNA delivery lend support to the feasibility of this approach^13^,^14^,^15^. Despite the fact that mRNA delivered *in vivo* are susceptible to highly active RNases that are ubiquitous in the extracellular space, we previously established a drug delivery system using polyplex nanomicelles to transport mRNA into target cells by preventing its degradation. Polyplex nanomicelles are based on the self-assembly of polyethylene glycol-polyamino acid (Poly{*N*-[*N*′-(2-aminoethyl)-2-aminoethyl]aspartamide}) block copolymer (PEG-PAsp(DET)), which possess a PEG outer layer and mRNA-containing core. This system provided excellent *in vivo* stability of mRNA under physiological conditions^16^,^17^. Furthermore, the stealth property provided by the polyplex nanomicelle surface, which is composed of dense PEG palisades, effectively prevented the inflammatory responses that are often caused by unfavorable immunogenicity of mRNA^16^.

In the present study, we present a disease-modifying strategy for OA with a focus on *in situ* mRNA delivery of a therapeutic, cartilage-anabolic transcription factor. Refinements in the molecular structure of polyplex nanomicelles improved the efficacy of *in vivo* mRNA introduction into the articular cartilage in mice. Then, the disease-modifying effects of delivery of a cartilage-anabolic transcription factor mRNA via the fine-tuned nanomicelles were examined in a mouse model of knee OA. The present study thus provides a new therapeutic strategy–namely, *in situ* delivery of mRNA encoding therapeutic transcription factors to ameliorate degenerative diseases.

## Results

### Successful mRNA delivery into the articular cartilage with polyplex nanomicelles

In addition to the PEG-PAsp(DET)-based nanomicelles mentioned in the introduction, we previously developed another type of block copolymer with a derivatized polyamino acid, poly(*N*-{*N* ’-[*N*”-(2-aminoethyl)-2-aminoethyl]-2-aminoethyl}aspartamide) PAsp(TET)^18^,^19^. PAsp(DET) and PAsp(TET) possess a similar structure of N-substituted polyaspartamides, but with different numbers of side chain aminoethylene repeats; the repeat number is 2 for PAsp(DET), and 3 for PAsp(TET). The slight difference in the repeat number made a significant difference in the intracellular fate of the exogenously-introduced mRNA^19^: PAsp(DET) efficiently transported mRNA into the cytoplasm, but the poor cytoplasmic stability led to facile degradation of mRNA, resulting in a less durable pattern of protein expression. In contrast, PAsp(TET) with its limited capability of endosomal escape eventually protected mRNA in the cytoplasm to induce sustainable expression from the mRNA.

**Figure 1:**
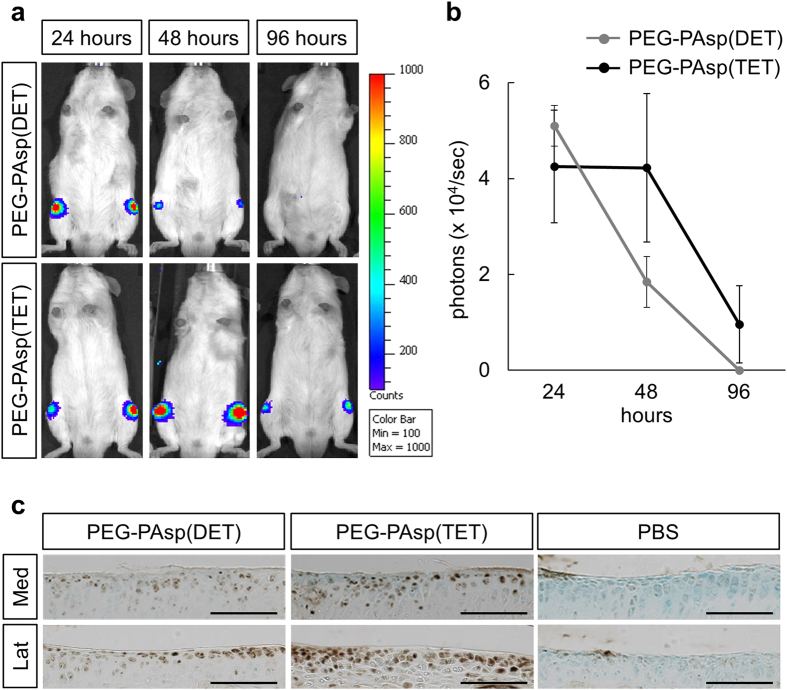
Protein expression from the reporter gene mRNA delivered into the articular cartilage using polyplex nanomicelles. (**a**) Expression of luciferase from mRNA that was delivered to mouse knee joints using polyplex nanomicelles. PEG-PAsp(DET) or PEG-PAsp(TET) polyplex nanomicelle solutions carrying 1 μg *luc*2 mRNA was injected once into intact knee joints of ICR mice. Luciferase expression was analyzed by the IVIS imaging system at the indicated hours after the injection. Four knees were used for the analysis, and representative images are shown. (**b**) Quantification of the IVIS data obtained in (**a**). (**c**) Expression of GFP from mRNA that was delivered to mouse knee joints using polyplex nanomicelles. PEG-PAsp(DET) or PEG-PAsp(TET) nanomicelle solutions carrying 1 μg *EGFP* mRNA was injected into intact knee joints. Phosphate buffered saline (PBS) was injected as a control. Samples were collected at 4 days after the injection and subjected to the immunohistochemical analysis using an anti-GFP antibody. Brown spots indicate positive staining. Counter staining was performed with methyl green. Med, medial site of the articular cartilage; Lat; lateral site of the articular cartilage. Scale bars, 100 μm.

Based on these facts, to identify the mRNA delivery system suitable for the articular cartilage, we newly synthesized a block copolymer of PEG-PAsp(TET) and compared its capacity for mRNA delivery into joint regions with that of PEG-PAsp(DET). The polyplex nanomicelles formed with PEG-PAsp(TET) or PEG-PAsp(DET) (see Methods) showed comparable physicochemical properties of around 50 nm size with almost neutral surface charge (see Supplementary Fig. S1 online). We injected nanomicelle solutions containing luciferase mRNA into intact mouse knee joints and analyzed luciferase expression from the delivered mRNA with an IVIS™ Imaging System. Both the PEG-PAsp(DET) and PEG-PAsp(TET) nanomicelles induced luciferase signals around the knee joints at as early as one day after the injection (Fig. 1a). The data suggest a trend that PEG-PAsp(DET) nanomicelles induced signals at 24 hours after the injection, although the signal then rapidly decreased and reached an undetected level at 4 days after the injection (Fig. 1a,b). In contrast, PEG-PAsp(TET) nanomicelle-injected group showed a trend of sustained luciferase expression for up to 4 days (Fig. 1a,b).

To further confirm mRNA delivery into articular chondrocytes, we next injected PEG-PAsp(DET) nanomicelles or PEG-PAsp(TET) ones containing enhanced green fluorescent protein (EGFP) mRNA into intact mouse knee joints and performed immunohistochemical analyses using an anti-GFP antibody at 4 days after the injection. GFP proteins were expressed in the superficial and middle zones of the articular cartilage (Fig. 1c). Consistent with luciferase data (Fig. 1a,b), more GFP-positive cells were observed in the PEG-PAsp(TET)-injected group than in the PEG-PAsp(DET)-injected one (Fig. 1c). Thus, both nanomicelles achieved mRNA delivery-mediated protein expression, but with different expression profiles, in the joint regions.

### Potential disease-modifying effects of mRNA delivery of a cartilage-anabolic transcription factor on OA

We then set out to examine whether our approach functioned in the manner of a DMOAD by using a mouse OA model. No existing method other than mRNA delivery could achieve the direct, vector-independent expressions of transcription factors in an exogenous manner. Therefore, to maximize the advantages of the approach, we chose a cartilage anabolic transcription factor, runt-related transcription factor (*Runx*) 1, as the mRNA delivered in this proof-of-concept study. *Runx1* has been shown to regulate chondrogenesis in embryos and adults^20^,^21^; we and others recently found that *Runx1*-mediated therapeutic effects of two different DMOAD candidate compounds, TD-198946 and kartogenin^22^,^23^. Regarding block copolymers for the mRNA delivery, we first chose PEG-PAsp(DET), which achieved high but transient expression of delivered mRNA in joint regions compared to PEG-PAsp(TET) (Fig. 1) and also permitted successful mRNA delivery into other tissues in our previous studies^16^,^17^.

We created OA in mouse knee joints by removing the medial collateral ligament and medial meniscus as previously described^24^. Then, beginning at one month after the operation, we injected PEG-PAsp(DET) nanomicelle solutions containing *EGFP* (control) or FLAG-tagged *RUNX1* (*RUNX1-FL*) mRNA into the OA joints once every 3 days for one month. The integrity of the protein translated from the *in vitro*-synthesized mRNA had been validated *in vitro* through several approaches (see Methods as well as Supplementary Fig. S2 online); we also confirmed that OA was moderately induced at the onset of the treatment compared with the sham-operated control, by evaluating OA severity using a histology-based OARSI scoring system^25^ (see Supplementary Fig. S3 online).

**Figure 2:**
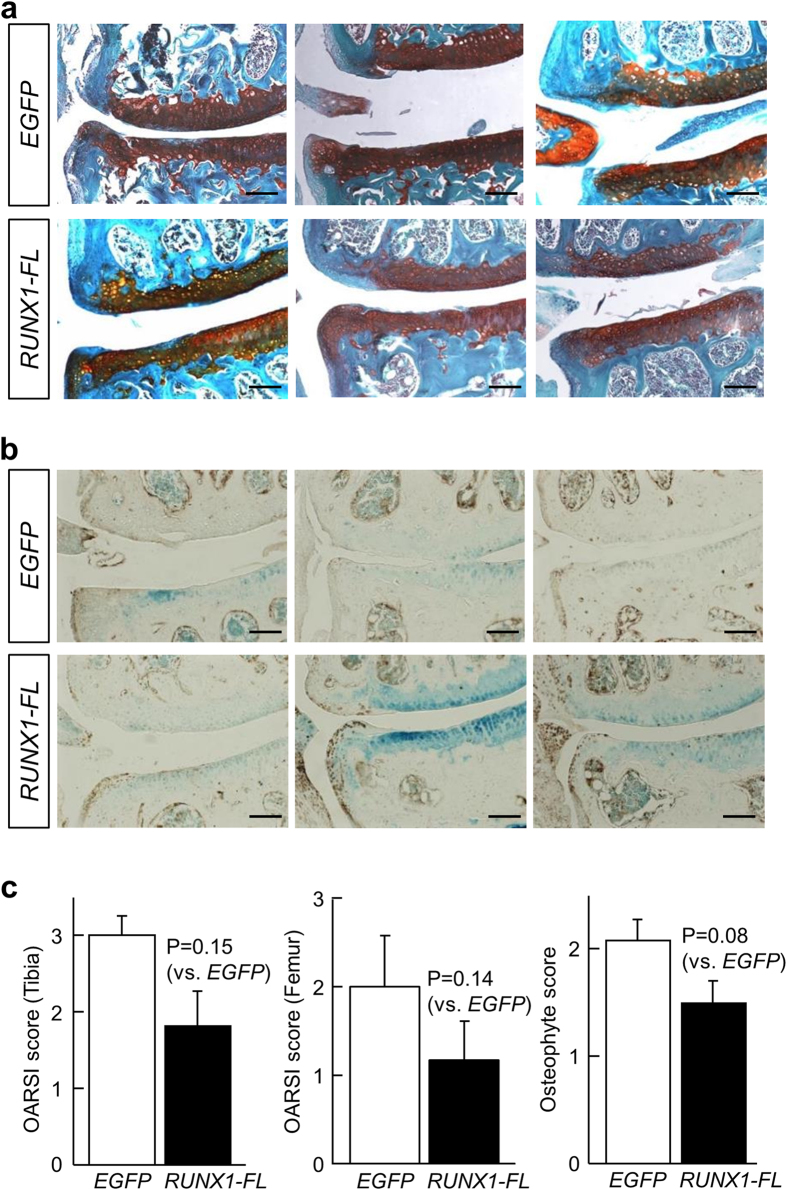
Histological analysis of mouse OA knee joints exposed to intra-articular injections of PEG-PAsp(DET) polyplex nanomicelles carrying *RUNX1-FL* or *EGFP* mRNA. (**a**) Images of representative sections stained with safranin-O after one month of intra-articular injection of 1 μg *RUNX1-FL* or *EGFP* mRNA-loaded PEG-PAsp(DET) polyplex nanomicelles. Scale bars, 100 μm. (**b**) Immunostaining on the serial sections of (**a**) using an anti-RUNX1 antibody. Brown spots indicate positive staining. Counter staining was performed with methyl green. Scale bars, 100 μm. (**c**) Scoring of OA status with the OARSI scoring system and the osteophyte scoring system. The score of normal cartilage is 0. Data are expressed as the mean ± SD (N = 10).

After the one-month series of injections, the control group showed typical signs of OA progression in histological sections stained with safranin-O, including osteophyte formation and cartilage degradation as evidenced by decreased thickness and staining intensity of the articular cartilage and disorganization of its structures (Fig. 2a). In contrast, the *RUNX1*-injected group showed a trend toward suppression of the OA phenotypes (compare *RUNX1-FL* with *EGFP* in Fig. 2a), suggesting that *RUNX1* mRNA delivery ameliorated OA; the potential effect was especially prominent in terms of osteophyte formation. Immunohistochemistry revealed that the expression of RUNX1 proteins was enhanced in the articular cartilage of the *RUNX1*-injected group, compared with the control group (Fig. 2b). The enhancement was more prominent in osteophyte-like regions than in other areas, suggesting a link between the suppressed OA phenotypes and RUNX1 proteins derived from the delivered mRNA in the *RUNX1*-injected group, although the immunohistochemistry could not discriminate exogenous from endogenous RUNX1. However, in quantitative assessments of OA progression (OARSI scoring system)^25^ and osteophyte formation^24^, there was no statistically significant difference between the control and *RUNX1*-injected groups, though a trend toward decrease in OA progression was observed (Fig. 2c).

### Improvement of disease-modifying effects of mRNA delivery of the cartilage-anabolic transcription factor on OA

**Figure 3:**
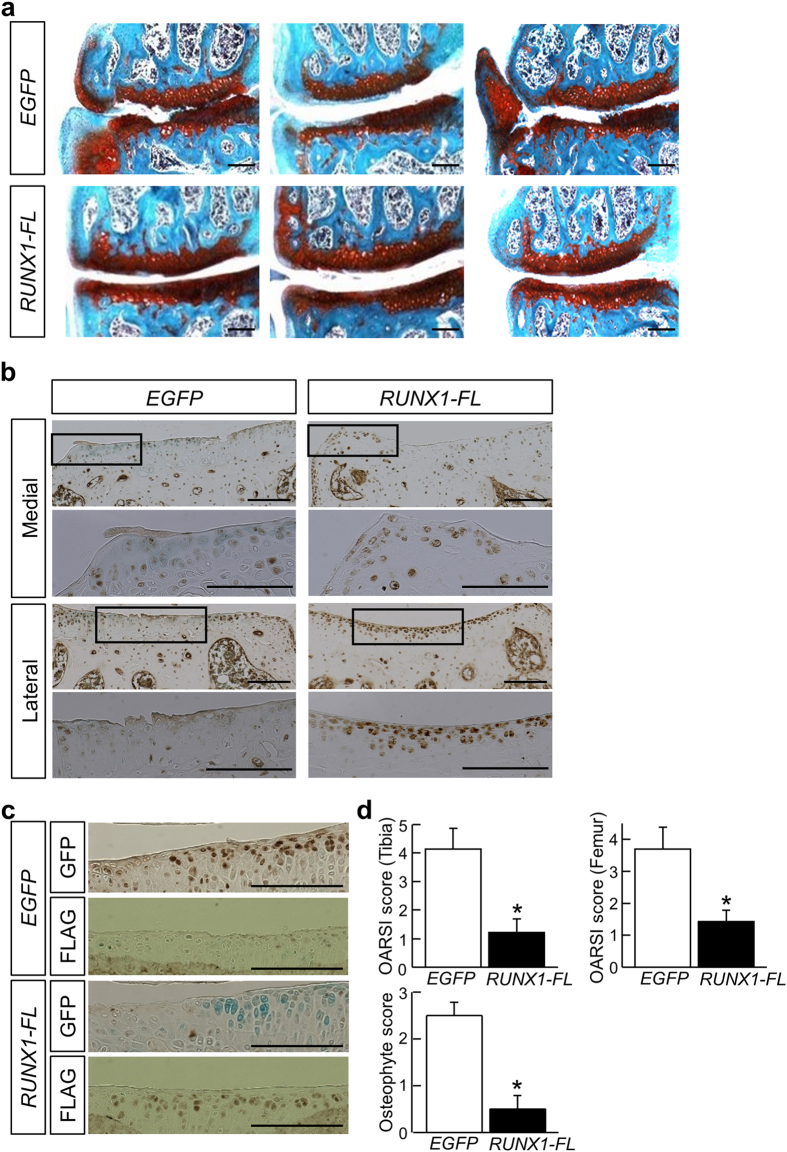
Histological analysis of mouse OA knee joints exposed to intra-articular injection of PEG-PAsp(TET) polyplex nanomicelles carrying *RUNX1-FL* or *EGFP* mRNA. (**a**) Images of representative sections stained with safranin-O after one month of intra-articular injection of 1 μg *RUNX1-FL* or *EGFP* mRNA-loaded PEG-PAsp(TET) polyplex nanomicelles. Scale bars, 100 μm. (**b**) Immunostaining of tibias using an anti-RUNX1 antibody on representative sections in (**a**). Brown spots indicate positive staining. Counter staining was performed with methyl green. Images of medial and lateral sites are shown. Medial sites were supposed to be more afflicted by the OA induction than lateral sites. Inset boxes indicate the magnified region shown below each image. Scale bars, 100 μm. (**c**) Immunostaining of tibias using anti-FLAG and anti-GFP antibodies on representative sections in (**a**). Brown spots indicate positive staining. Counter staining was performed with methyl green. Scale bars, 100 μm. (**d**) Scoring of OA status with both the OARSI scoring system and the osteophyte scoring system. The score of normal cartilage is 0. Data are expressed as the mean ± SD (N = 10). ^*^P < 0.05 versus the *EGFP* mRNA-injected group.

Because the protein expression by the PEG-PAsp(TET) nanomicelles in the joint regions was prolonged compared to that by the PEG-PAsp(DET) ones (Fig. 1), we next attempted to improve the disease-modifying effects of *RUNX1* mRNA delivery by using PEG-PAsp(TET). We adopted the same OA model and injection protocol as used in the earlier studies with PEG-PAsp(DET). Histological analyses with safranin-O staining revealed that OA progression was suppressed in the *RUNX1*-injected group compared with the control group, in terms of both cartilage degradation and osteophyte formation (Fig. 3a). No obvious inflammatory symptoms, such as outgrowth of the synovial membrane or infiltration of inflammatory cells, were observed on the histological sections (Fig. 3a). As was the case in the earlier study, overall RUNX1 protein expression in the articular cartilage was enhanced in the *RUNX1*-injected group (Fig. 3b). In addition, the results of immunohistochemistry for GFP and FLAG supported the idea that exogenously introduced GFP and RUNX1-FL were expressed in specific regions of the articular cartilage in the control group and the *RUNX1*-injected group, respectively; namely, the exogenous proteins were expressed in the superficial and middle zones of the articular cartilage (Fig. 3c). Consistent with the histological observations, histology-based scoring demonstrated that both overall OA progression and osteophyte formation were significantly suppressed in the *RUNX1*-injected group compared with the control group (Fig. 3d). Thus, PEG-PAsp(TET)-mediated mRNA delivery of *RUNX1* induced exogenous protein expression in the OA cartilage, and thereby exerted significant disease-modifying effects on OA in the mouse model.

### Characterization of disease-modifying effects of the present strategy on OA

**Figure 4:**
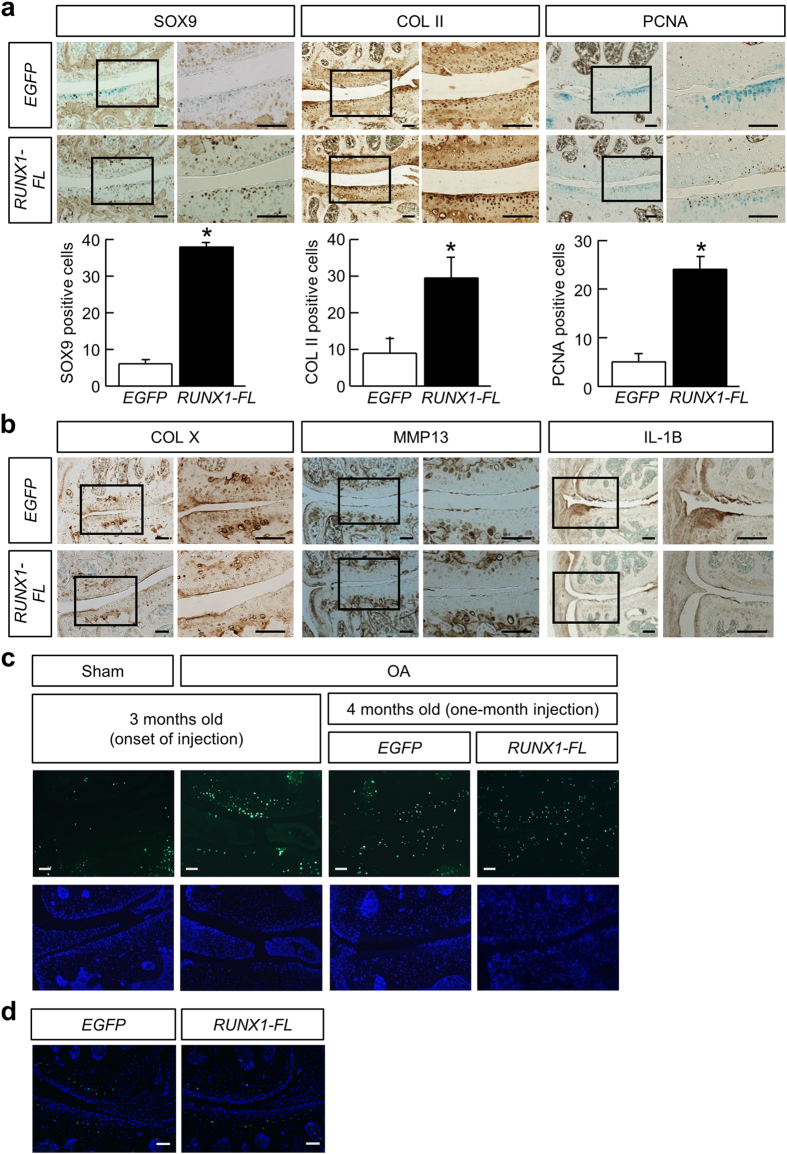
Immunohistochemical analyses of mouse OA knee joints exposed to intra-articular injection of PEG-PAsp(TET) polyplex nanomicelles carrying *RUNX1-FL* or *EGFP* mRNA. (**a**) Immunostaining using anti-Sox9, anti-type II collagen, and anti-PCNA antibodies on sections after one month of intra-articular injection of 1 μg *RUNX1-FL* or *EGFP* mRNA-loaded PEG-PAsp(TET) polyplex nanomicelles. Brown spots indicate positive staining. Counter staining was performed with methyl green. Inset boxes indicate the magnified region shown on the right of each image. Scale bars, 100 μm. Quantitative data of stained cells are shown below each image. Data are expressed as the mean ± SD (N = 3). ^*^P < 0.05 versus the *EGFP* mRNA-injected group. (**b**) Immunostaining using anti-type X collagen, anti-MMP13, and anti- IL-1ß antibodies on sections after one-month intra-articular injection of 1 μg *RUNX1-FL* or *EGFP* mRNA-loaded PEG-PAsp(TET) polyplex nanomicelles. Brown spots indicate positive staining. Counter staining was performed with methyl green. Inset boxes indicate the magnified regions shown on the right of each image. Scale bars, 100 μm. (**c**) TUNEL staining of the indicated groups. Green spots indicate apoptotic cells in upper panels. DAPI images of the identical sections are also shown at the bottom of each section. Scale bars, 100 μm. (**d**) TUNEL staining of the lateral side of knees analyzed in (**c**). Scale bars, 100 μm.

We investigated the molecular basis of the observed disease-modifying effects of the present strategy. Expression of SOX9, a master transcription factor for the specification and maintenance of cartilage in both embryos and adults, was increased in articular chondrocytes of the *RUNX1*-injected group compared with the control group (SOX9 in Fig. 4a). Type II collagen, a major cartilage matrix protein, was also increased in the *RUNX1*-injected group compared with the control (COL II in Fig. 4a). This result is consistent with the increased expression of SOX9, since both genetic and biochemical studies have established that SOX9 plays crucial roles in expression of the *COL2A1* gene^26^,^27^,^28^,^29^. Moreover, expression of the proliferating nuclear antigen (PCNA) was upregulated in articular chondrocytes of the *RUNX1*-injected group (PCNA in Fig. 4a). Thus, both differentiation and proliferation of articular chondrocytes were augmented by the *RUNX1* injection.

Given that the late stage of OA mimics the process of endochondral ossification, we next investigated whether the expression of hypertrophic chondrocyte markers, type X collagen and matrix metalloprotainase (MMP) 13, was altered upon the *RUNX1* injection compared with the control. As shown in Fig. 4b, there was no discernible change in those expressions between the two groups, suggesting that the present strategy does not modify the process in OA. We then investigated expression of interleukin-1 beta (IL-1 beta), since IL-1 beta is a major proinflammatory factor in pathogenesis of OA^30^,^31^. IL-1 beta was decreased in the *RUNX1-FL*-injected group compared with the control group (Fig. 4b).

Chondrocyte apoptosis is known to be increased in OA progression compared with the healthy cartilage, and the number of apoptotic cells in the articular cartilage is significantly higher in OA patients^32^,^33^. When compared with the sham-operated group, TdT-mediated dUTP nick end labeling (TUNEL)-positive cells were increased in the articular cartilage of mice that were exposed to OA induction for one month, i.e., at the onset of intra-articular injections (Fig. 4c). The pattern of TUNEL-positive chondrocytes was not largely changed even after the one-month injection of either *EGFP* or *RUNX1* (Fig. 4c). In addition, the *RUNX1* injection had little effects on apoptosis of articular chondrocytes at the less afflicted, lateral sites of OA-operated knees (Fig. 4d). Thus, the *RUNX1* injection is unlikely to alter the apoptotic state of either afflicted or less afflicted chondrocytes in OA joints, and our nanomicelle-mediated mRNA delivery has little nanoparticle-associated toxicity^34^,^35^ in the articular cartilage.

## Discussion

The present study has three major findings. First, a sophisticated mRNA carrier, polyplex nanomicelle, realized the intra-articular administration of mRNA; it achieved efficient protein expression from the exogenous mRNA in the articular cartilage. Second, the delivery of mRNA encoding the cartilage anabolic transcription factor *RUNX1* successfully suppressed the progression of OA in mouse knee joints with little toxicity or inflammatory responses. Third, the suppression of OA progression was accompanied with the enhanced expression of cartilage anabolic and proliferation markers in articular chondrocytes. Based on these findings, we propose a new strategy for OA treatment by mRNA-based therapeutics, in which the “therapeutic” transcriptional factor mRNA is delivered into the joints to directly regulate the cartilage anabolism.

Gene therapy, while drawing much attention as a potential approach for OA^36^,^37^, is still less common than other strategies due to its limited efficacy and safety. Viral vectors have the potential to introduce genes of interest at high capacity^38^, but their clinical application to non-lethal diseases such as OA remains problematic due to safety concerns. Plasmid DNA (pDNA) generally has a very low capacity for gene introduction in non-dividing cells, because it needs to be transported into the nucleus: the nuclear membrane is a formidable intracellular barrier for non-viral gene delivery^39^. In contrast, mRNA-based strategies have the potential to be a more direct and efficient way to express proteins of interest inside the cells without the above issues associated with viral vectors or pDNA. Unlike viral vectors or pDNA, mRNA is never integrated into the genome and does not need to enter the nucleus to express its coding protein, enabling transfection of non-dividing cells and early onset of protein expression as well as eliminating the risk of insertional mutagenesis^11^,^40^. We previously demonstrated that polyplex nanomicelles composed of mRNA and PEG-PAsp(DET) block copolymer had a high capacity for *in vivo* mRNA delivery in neural cells, most of which are mature non-dividing cells, and delivery of neurotrophic factor mRNA using the system ameliorated neurological dysfunction in rodent models^16^,^17^. In addition to the stability of nanomicelles under physiological conditions, the nanomicelles effectively suppressed immune responses that could be induced by exogenous mRNA, likely due to the shielding effect of PEG to avoid mRNA recognition by Toll-like receptors on host immune cells. From the economical standpoint, the cost for mRNA preparation is higher than that for DNA preparation at present. However, the cost for *in-vitro* transcription can be drastically reduced by the benefit from a larger production scale; the process, which relies just on enzymatic reactions, can be performed in a similar manner independently of nucleic acid sequences once DNA templates are prepared. Moreover, the production cost of mRNA would be much lower than that of recombinant proteins, which can also increase the clinical feasibility of mRNA-based therapies^11^.

In the present study, both PEG-PAsp(DET) and PEG-PAsp(TET) successfully induced protein expression from delivered mRNA with distinct expression profiles in joint regions. However, when combined with a therapeutic transcription factor mRNA, they had different therapeutic effects on OA; PEG-PAsp(TET)-mediated prolonged expression of the transcription factor is likely to be more suitable for OA treatment than PEG-PAsp(DET)-mediated high but transient expression. Together with our previous findings that PEG-PAsp(DET)-mediated mRNA delivery was successful in treating neurological dysfunctions^16^,^17^, these results also suggest that the suitability of molecular structures of block copolymers is dependent on the target cells and/or modes of action of the delivered factors (secreted from target cells or acting inside of the cells). The structural features of joints as well as the properties of polyplex nanomicelles may contribute to the sustainability of protein expression and therapeutic effects; articular capsules, closed spaces created by synovial membranes and filled with a synovial fluid, may increase the delivery rate of mRNA to target cells. In addition, cationic carriers such as Lipofectamine^TM^ 2000 showed no protein expression by intraarticular injection (data not shown). Given that the cationic surface of the carriers promptly form aggregates after the injection^41^, the stealth property of nanomicelles due to the PEG surface would be essential for mRNA delivery even when injecting into closed spaces. However, multiple administrations of mRNA-containing nanomicelles are still required to maintain the therapeutic effects for one month, which is a potential limitation of the present strategy. Sustained release of the mRNA-containing nanomicelles in the joint space could be achieved by using injectable biomaterials as a vehicle for the nanomicelles. We recently developed an injectable, non-swellable hydrogel, in which the osmotic pressure-induced swelling, a major limitation of conventional hydrogels for *in vivo* use, was cancelled by the controllable collapse of an installed thermoresponsive polymer unit at a certain critical temperature^42^. Such hydrogels would be strong candidates for vehicles of the nanomicelles in the joint space.

Clinical trials have been performed on a variety of agents as DMOAD candidates; several agents are in phase II trials or beyond. They can be classified into several categories depending on their modes of action and targets: anabolic agents for subchondral bones (bone morphogenetic protein 7- BMP7) and for cartilage (fibroblast growth factor 18- FGF18), anti-catabolic agents for subchondral bones (calcitonin) and for cartilage (iNOS inhibitors), and agents that have both anabolic and anti-catabolic effects (avocado-soybean unsaponifiables)^7^. Despite the vast body of knowledge on cartilage anabolic factors, very few cartilage anabolic agents have yet been identified. Cartilage anabolic agents would be expected to extend the range of therapeutic strategies for patients with OA, even permitting the treatment of progressed OA. Therefore, in the present proof-of-concept study, we chose the cartilage anabolic transcription factor Runx1, and delivered its mRNA into mouse knee joints for OA treatment.

*Runx1* is initially expressed in undifferentiated mesenchyme during endochondral ossification in mice^43^,^44^. The expression is maintained in mitotic chondrocytes that express type II collagen and excluded from post-mitotic hypertrophic chondrocytes^43^. Deletion of *Runx1* from *Prrx1*-positive skeletal progenitors causes a delay in sternum development, whereas *Runx1* deficiency in *Col2a1*-expressing chondrocytes does not affect skeletal development^20^. Consistent with these *in vivo* data, we previously reported that Runx1 activated transcription of the *Col2a1* gene by binding to a Runx motif present at its 5′ flanking region^22^. Kimura *et al.* proposed that Runx1 induced the expressions of Sox5 and Sox6, cooperative factors of Sox9^20^. These data indicate that Runx1 plays a role in promoting early differentiation of chondrocytes, i.e., cartilage anabolic function; together with the fact that Sox5, 6, and 9 are master regulators of *Col2a1*, these findings also suggest the presence of a transcriptional network between Runx1, Sox5, Sox6, and Sox9 that specifies *Col2a1*-expressing chondrocytes.

In addition to the above findings in cartilage development, several lines of evidence in adults provide a further rationale for using Runx1 in OA treatment. *Runx1* is expressed in the articular cartilage^21^,^22^. Importantly, positive regulation of Runx1 expression and activity mediates therapeutic effects of two recently identified DMOAD candidates, TD-198946^22^ and kartogenin^23^. In the study that linked TD-198946 with Runx1, we observed loss of Runx1 expression in mouse and human OA cartilage^22^, suggesting that Runx1 plays a protective role in OA progression. Taking these findings together, one could further imagine that the protective role of Runx1, if any, is mediated by its anabolic function in the articular cartilage. Our present data are in harmony with this notion. The suppression of OA progression by *RUNX1* mRNA delivery was accompanied by enhanced expression of Sox9 and its transcriptional target type II collagen. Thus, we provided *in vivo* data that potentially support the therapeutic efficacy of the cartilage anabolic function of Runx1 in cartilage disorders. In addition, we consider two potential approaches to enhance the therapeutic efficacy of our present strategy: combinatorial use of Runx1 with co-factors that enhance the function of Runx1 itself or that with other cartilage anabolic transcription factors. Core binding factor beta (Cbfb), a major co-factor for Runx family proteins, would be a logical candidate for the first approach. Cbfb forms heterodimers with Runx proteins; the heterodimerization enhances transcriptional activities of Runx proteins by stabilizing them as well as augmenting their DNA binding abilities^45^,^46^. Sox9 would be a candidate as a cartilage anabolic transcription factor that cooperatively suppresses OA progression with Runx1, given its chondrogenic actions^27^ as well as the potential transcriptional network between Runx1, Sox5, Sox6, and Sox9 that we discussed earlier.

Which stage of OA does the exogenous *RUNX1* primarily ameliorate? The anabolic and mitotic functions of Runx1 would protect the articular cartilage from initial degradation, which is partly supported by our data on the OARSI scoring. Osteophyte formation was also suppressed in the *RUNX1*-injected group, suggesting that Runx1 ameliorated the late-onset, endochondral ossification-like process as well. However, no discernible change in the expressions of hypertrophic chondrocyte markers was observed between the control and *RUNX1*-injected group. It is possible that the primary action of Runx1 on the initial degradation of the articular cartilage secondarily suppressed the subsequent osteophyte formation. We are currently verifying the direct actions of Runx1 on osteophyte formation and chondrocyte hypertrophy, and will describe our findings in a future report.

The articular chondrocyte populations to which mRNA is delivered may also account for its therapeutic mechanisms. Protein expression from the delivered mRNA was achieved only in the superficial and middle zones, but not the deep zone, of the articular cartilage, indicating that this strategy had no effect on chondrocytes in the deep zone. From this standpoint, the finding that there was no change in the expression of hypertrophic chondrocyte markers in the deep zone may be considered reasonable, even if Runx1 itself has some roles in chondrocyte hypertrophy. The importance of the superficial zone in OA progression has been demonstrated in association with protective roles of Prg4 against OA^47^,^48^. Given that Runx1 as well as Prg4 are expressed in the superficial zone chondrocytes and their expressions are sensitive to mechanical loading^21^, the delivered Runx1 may cooperate with Prg4 to protect the superficial zone chondrocytes from mechanical stress in the early phase of OA. Recent data on the fates of *Prg4*-expressing cells^49^ further suggest that the protected *Prg4*-expressing cells in the superficial zone extend to deeper regions and augment the net resistance of the articular cartilage to mechanical loading. The deepest cells seem not to contribute here, since they are not descendants of the superficial zone chondrocytes that have expressed *Prg4* in adults^49^, which also conforms with our therapeutic model, as mentioned earlier in this paragraph. In addition, we observed an increase in PCNA-positive cells during *RUNX1* mRNA delivery-mediated OA suppression. Thus, the positive role of Runx1 in proliferation of articular chondrocytes is also likely to underlie its therapeutic effects on OA progression, contributing to the augmentation of the net resistance of the articular cartilage to the mechanical loading. Consistent with this idea, Leblanc *et al.* showed overlapping expression of Runx1 and proliferation markers including PCNA and Ki-67 in human OA cartilage as well as reduction of a proliferation marker in *Runx1*-deleted mouse embryonic fibroblasts^21^. Taken together, Runx1 is likely to suppress OA progression by acting on remaining chondrocytes, and therefore to be more effective on the early stage of OA than on the later one, in which the articular cartilage is fully eroded.

The decreased expression of IL-1 beta in the *RUNX1*-injected group suggests that Runx1 may suppress the inflammatory response in OA progression, although the relationship between Runx1 and IL-1 beta is unclear. *RUNX1* is a susceptibility gene for rheumatoid arthritis (RA), but the susceptibility has been explained by the association of single nucleotide polymorphisms in the Runx1-binding site of the *SLC22A4* region with RA^50^,^51^. Our data may suggest that *RUNX1* mRNA delivery has potential for the treatment of not only OA but also RA, the pathogenesis of which is mediated by IL-1 beta^52^, although the ability of the present strategy to deliver mRNA to the synovial membrane must be further investigated. Thus, we anticipate that the direct delivery of therapeutic mRNAs into the articular cartilage will lead to disease-type-specific treatments for different types of arthritis. More importantly, this approach will enable us to directly apply basic findings on disease-protective or tissue-regenerating factors to the treatment of degenerative diseases, even when they are intracellular molecules such as transcription factors.

## Methods

### Chemicals

β-Benzyl-L-aspartate *N*-carboxyanhydride (BLA-NCA) was purchased from Chuo Kasei Co. Ltd. (Osaka, Japan). α-methoxy-ω-amino poly(ethylene glycol) (PEG-NH_2_) (Mw 23,000) was obtained from Nippon Oil and Fats (Tokyo, Japan); diethylenetriamine (DET), triethylenetetramine (TET), tris-(hydroxymethyl)aminomethane (Tris), and 2-[4-(2-hydroxyethyl)-1-piperazinyl]ethanesulfonic acid (HEPES) were purchased from Wako Pure Chemical Industries, Ltd. (Osaka, Japan). DET and TET were used after conventional distillation.

### Synthesis of PEG-PAsp(DET) and PEG-PAsp(TET)

A PEG-block-poly(β-benzyl-L-aspartate) (PEG-b-PBLA) was synthesized by the ring-opening polymerization as previously reported^53^. Briefly, the polymerization of BLA-NCA was initiated from the terminal primary amino group of PEG-NH_2_ to obtain PEG-b-PBLA, followed by aminolysis reaction to introduce DET or TET into the side chain of PBLA. The synthesized block polycations were determined to have a narrow unimodel molecular weight distribution (M_w_/M_n_ = 1.04) based on gel permeation chromatography (GPC) measurement. The polymerization degrees of PEG-PAsp(DET) and PEG-PAsp(TET) were calculated as 66 (DET) and 65 (TET) by using ^1^H NMR measurement (JEOL EX300 spectrometer, JEOL, Tokyo, Japan).

### Characterization of polyplex nanomicelles

Particle size and polydispersity (PDI) of nanomicelles were measured by dynamic light scattering (DLS) measurements using a Zetasizer Nano ZS (Malvern Instruments Ltd., Worcestershire, UK) with a He-Ne laser (λ = 633) at a detection angle of 173° and a temperature of 25 °C. Nanomicelle samples were added into a small glass cuvette (volume: 12 μL) ZEN2112. The obtained data from the ratio of decay in the photon correlation function were calculated by the Stokes-Einstein equation. Zeta potential of nanomicelles was measured using the Zetasizer Nano ZS. Nanomicelle samples were added into a folded capillary cell DTS 1060. The zeta potential of nanomicelles was calculated by the Smoluchowski equation.

### Preparation of mRNA

We cloned a 3xFLAG-tagged human *RUNX1* ORF sequence (*RUNX1-FL*) flanked by *Xho*I and *Bal*I/*Sma*I into pUC57 vectors. The *RUNX1-FL* sequence was cloned into pSP73. T7 promoter-mediated transcription was performed on the linearized pSP73-*RUNX1-FL* using an mMESSAGE mMACHINE T7 Ultra Kit (Ambion, Carlsbad, CA), followed by polyadenylation using a poly(A) tail kit (Ambion). Transcribed mRNA was purified with an RNeasy Mini Kit (Qiagen, Hilden, Germany). The concentration and integrity of the *in-vitro*-transcribed mRNA were determined by a Nanodrop 2000 (Thermo Fisher Scientific, Waltham, MA) and 2100 Bioanalyzer (Agilent Technologies, Santa Clara, CA), respectively. mRNA of luciferase (*luc2* in pGL4.10; Promega, Madison, WI) and enhanced green fluorescent protein (EGFP) were also prepared as described above after cloning into the pSP73 vectors.

### Preparation of mRNA-loaded polyplex nanomicelles

Block copolymers (PEG-PAsp(DET) or PEG- PAsp(TET)) and mRNA were separately dissolved in 10 mM HEPES buffer (pH 7.3). The concentration of mRNA was set at 75 μg/mL, and those of the block copolymers were adjusted to obtain an N/P ratio (the residual molar ratio of the polycations amino groups to the mRNA phosphate groups) of 8. Solutions of the block copolymers and mRNA were mixed at a ratio of 1:2 to obtain polyplex solutions with a final mRNA concentration of 50 μg/mL. Lipoplex with mRNA was prepared using Lipofectamine^TM^ 2000 (Life Technologies, Carlsbad, CA) according to the manufacturer’s protocol.

### Assessment of luciferase expresion in mouse knee joints

Twenty microliters of PEG-PAsp(DET) or PEG-PAsp(TET) polyplex nanomicelle solution containing 1 μg *luc*2 mRNA were injected into intact knee joints of 10-week-old ICR mice (purchased from Charles River, Yokohama, Japan) using a 30 G syringe under anesthesia. Luciferase expresion was observed at various time points (24, 48, and 96 hours) using an IVIS™ Imaging System (Xenogen, Alameda, CA) after intravenous injection of 1.5 mg D-luciferin (100 μL of 15 mg/mL solution).

### Mouse OA model and intra-articular injection

Left knee joints of 8-week-old C57BL/6J mice (purchased from Charles River) were used for the OA model. The model was established as previously described^24^. Briefly, under general anaesthesia, the medial collateral ligament was transected, and the medial meniscus was removed using a surgical microscope. One month after the surgery, 20 μL of nanomicelle solution containing 1 μg mRNA was injected into the knee joints of mice using a 30 G syringe under reanaesthesia. All the animal experimental protocols were approved by the Institutional Animal Care and Use Committee of the University of Tokyo; all the experiments were carried out in accordance with the approved guidelines.

### Histological analysis

Eight weeks after the OA surgery (after one-month intra-articular inection), the mice were euthanized and subjected to perfusion fixation with 4% paraformaldehyde (PFA)/phosphate buffered saline (PBS). Knee joints were collected, post-fixed with 4% PFA/PBS overnight, and decalcified with 0.5 M ethylenediaminetetraacetic acid (EDTA)/PBS. Decalcified samples were embedded in paraffin and sectioned frontally at a thickness of 7-μm. OA severity was assessed by the OARSI scoring system (for tibias and femurs)^25^ and the osteophyte scoring system^24^ on sections stained with safranin-O. Safranin-O staining was performed according to the standard protocol. Briefly, after deparaffinization and hydration, sections were stained with fast green (FSF) solution for 5 minutes, rinsed quickly in 1% acetic acid, and stained with 0.1% safranin-O solution for 5 minutes. Sections were then dehydrated, cleared with a series of alcohol and xylene, and mounted. For immunohistochemistry, sections were incubated with antibodies to Flag (1:100, F1804, M2; Sigma-Aldrich, St. Louis, MO), GFP (1:500, ab290; Abcam, Cambridge, MA), Runx1 (1:100, ab23980; Abcam), Sox9 (1:200, sc20095; Santa Cruz Biotechnology, Santa Cruz, CA), and PCNA (1:1000, 2586s; Cell Signaling Technology, Danvers, MA) combined with a CSAII Biotin-free Tyramide Signal Amplication System (Dako, Glostrup, Denmark). Staining with antibodies to type X collagen (1:500, LB-0092; LSL-Cosmo Bio Co.,Ltd., Tokyo, Japan), type II collagen (1:500, LB-1297; LSL-Cosmo Bio Co., Ltd.), IL-1 beta (1:100, sc7884; Santa Cruz Biotechnology), and MMP-13 (1:500, mab13426; Millipore, Billerica, MA) was performed according to standard protocols. Antigen-antibody complex was visualized by the reaction of peroxidase and diaminobenzodine (DAB), which gave brown signal. Sections were counterstained with methyl green. Quantification of immunostaining data is performed by counting stained cells in 400 × 100 μm regions of medial zone of tibia plateau for each section, as previously described^54^. The TUNEL assay was performed using a TUNEL detection kit (Takara Shuzo, Kyoto, Japan) according to the manufacturer’s instructions. Briefly, knee joint sections were incubated with 15 mg/mL of proteinase K for 15 min at room temperature and then washed with PBS. The sections were immersed in TdT Enzyme diluted with Labeling Safe Buffer (provided in the kit) and then incubated for 90 min at 37 °C in a humidified atmosphere. After washing with PBS, the slides were examined by fluorescence microscopy.

### SDS-PAGE and western blotting

HEK293 cells were obtained from the Riken Cell Bank (Tsukuba, Japan). One million cells were seeded on 10-cm dishes and cultured in high glucose Dulbecco’s modified Eagle’s medium (DMEM, Sigma-Aldrich) containing 10% fetal bovine serum (Sigma-Aldrich) and 1% penicillin/streptomycin (Sigma-Aldrich). Twenty-four micrograms of plasmid DNA or *in vitro*-synthesized mRNA was transfected with 60 μL lipofectamine 2000. Fluorescent images for GFP were obtained by an Axiovert 100 M microscope (Carl Zeiss, Oberkochen, Germany). Cells were lysed at 8 or 24 hours after transfection. *In vitro* cell-free transcription/translation was performed using a TNT Quick Coupled Transcription/Translation System (Promega) according to the manufacturer’s protocol. Cell lysate and *in vitro* transcription/translation reaction mixtures were fractionated by SDS–PAGE with Novex 4–20% Tris-Glycine Mini Protein Gel (Life Technologies), and transferred onto nitrocellulose membranes (Bio-Rad, Hercules, CA). After being blocked in 6% milk/Tris buffered saline with 0.1% Tween (TBST), the membranes were incubated with a horseradish peroxidase (HRP)-conjugated anti-Flag M2 mouse monoclonal antibody (1:1000, A8592; Sigma-Aldrich) or an anti-Runx1 rabbit antibody (1:100, sc28679; Santa Cruz Biotechnology) overnight. For anti-Runx1 antibodies, membranes were incubated with secondary antibodies (HRP-conjugated goat anti-rabbit IgG, Promega) at a dilution of 1:10,000 for 1 hour. Immunoreactive signals were visualized with an ECL Plus western blotting detection reagent (GE Healthcare, Little Chalfont, UK) according to the manufacturer’s instructions, and analyzed with a lumino image analyzer (Model LAS-4000 Mini; Fujifilm, Tokyo, Japan) coupled with image analysis software (Multi Gauge Ver. 3.0; Fujifilm).

### Statistical analysis

Data are expressed as the mean ± SD. Statistical analysis was performed with the Mann-Whitney U test. p values < 0.05 were considered significant.

## Additional Information

**How to cite this article**: Aini, H. *et al.* Messenger RNA delivery of a cartilage-anabolic transcription factor as a disease-modifying strategy for osteoarthritis treatment. *Sci. Rep.* **6**, 18743; doi: 10.1038/srep18743 (2016).

## Supplementary Material

Supplementary Information
