# Supplementary material for: Messenger RNA delivery of a cartilage-anabolic transcription factor as a disease-modifying strategy for osteoarthritis treatment
Source: Sci Rep. 2016 Jan 5;6:18743. doi: 10.1038/srep18743 (PMC4700530; doi:10.1038/srep18743)
Supplement: Supplementary Information [file srep18743-s1.pdf]

Messenger RNA delivery of a cartilage-anabolic transcription factor as a disease-modifying strategy for osteoarthritis treatment

Hailati Aini, Keiji Itaka, Ayano Fujisawa, Hirokuni Uchida, Satoshi Uchida, Shigeto Fukushima, Kazunori Kataoka, Taku Saito, Ung-il Chung, Shinsuke Ohba

Supplemantary Figures

Supplementary Figure 1 Size distribution, particle size, polydispersity (PDI), and zeta potential of nanomicelles prepared from PEG-PAsp(DET) or PEG-PAsp(TET)
Supplementary Figure 2 Confirmation of protein expressions from *in vitro*-synthesized mRNA
Supplementary Figure 3 Confirmation of OA induction in the mouse model used in this study

a

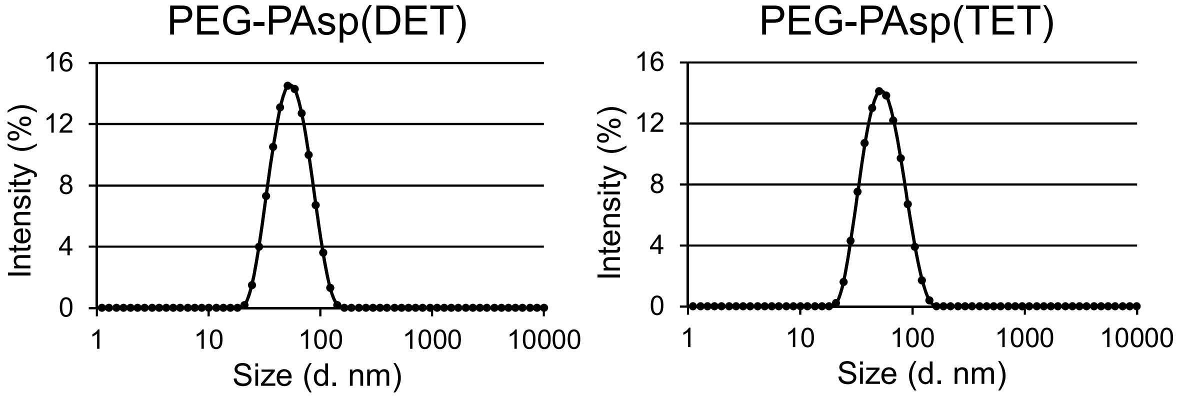

b

	Size (nm)	PDI	ζ-potential
PEG-PAsp(DET)	52.83 ±1.4	0.162 ±0.019	0.071 ±0.159
PEG-PAsp(TET)	50.45 ±1.4	0.145 ±0.012	0.097 ±0.037

Supplementary Figure 1. Size distribution, particle size, polydispersity (PDI), and zeta potential of nanomicelles prepared from PEG-PAsp(DET) or PEG-PAsp(TET). (**a**) Size distribution of nanomicelles measured by dynamic light scattering (DLS). (**b**) Average particle size, polydispersity (PDI), and zeta potential of nanomicelles with standard deviation (N = 4).

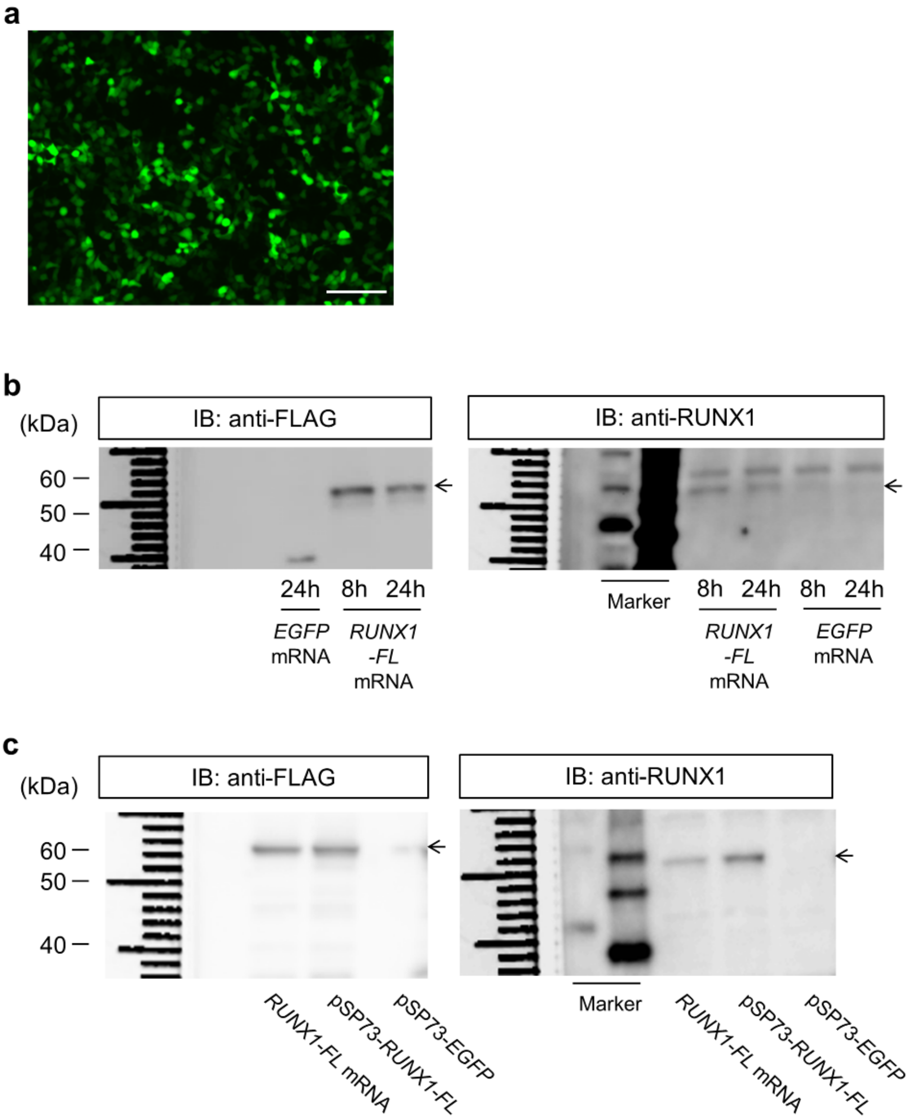

Supplementary Figure 2. Confirmation of protein expressions from the *in vitro*-synthesized mRNA. (**a**) GFP fluorescence in HEK293 cells transfected with the *in vitro*-synthesized *EGFP* mRNA. Twenty-four hours after lipofection of *EGFP* mRNA, the images were taken by fluorescent microscopy. Scale bar, 100 µm. (**b**) Protein expression of RUNX1-FL in HEK293 cells transfected with the *in vitro*-synthesized *RUNX1-FL* mRNA. Whole cell lysates were obtained at 8 or 24 hours after lipofection of the mRNA. Cells transfected with *EGFP* mRNA were used as a negative control. Immunoblotting was performed using an anti-FLAG antibody and an anti-RUNX1 antibody. Arrows indicate bands of RUNX1-FL of the expected size. (**c**) Validation of RUNX1-FL protein translated from the *in vitro*-synthesized *RUNX1-FL* mRNA in the cell-free system. RUNX1-FL proteins were synthesized directly by translation of the *in vitro*-synthesized *RUNX1-FL* mRNA or via transcription from pSP73-*RUNX1-FL* vectors and subsequent translation. Reaction mixtures were analyzed by immunoblotting using an anti-FLAG antibody and an anti-RUNX1 antibody. The reaction mixture from pSP73-*EGFP* was used as a negative control. Arrows indicate bands of RUNX1-FL of the expected size.

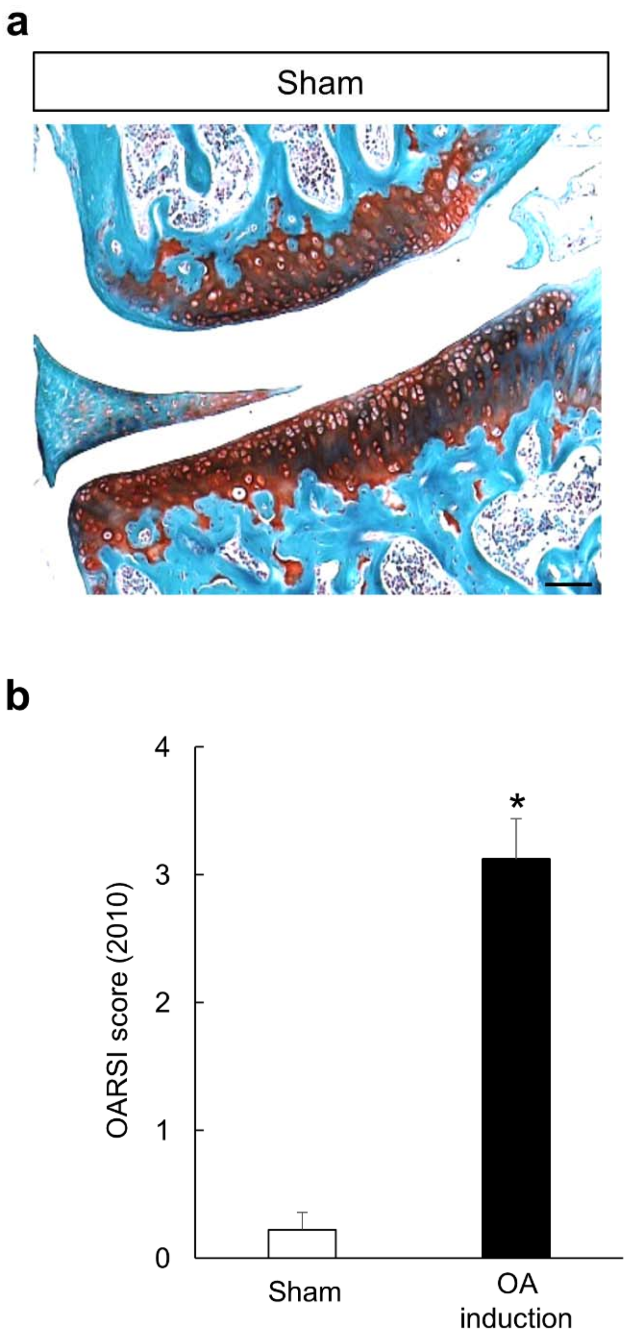

Supplementary Figure 3. Confirmation of OA induction in the mouse model used in this study. (**a**) Representive images of safranin-O staining of sections from the sham-operated and the OA-operated groups. A month after the OA surgery, all knee joints were histologically analyzed by safranin-O staining. The circle indicates an osteophyte-like lesion, and asterisks indicate the degeneration of the articular cartilage as evidenced by decreased intensity of staining. (**b**) Scoring of the OA state by OARSI scoring system on sections stained with the safranin-O. Data are expressed as the mean ± S.D. (N = 5). $^{*}P < 0.05$ vs. the sham operated group. Scale bars, 100 μm.
